# Evaluation of ethanol vortex ELISA for detection of bovine tuberculosis in cattle and deer

**DOI:** 10.1186/1746-6148-10-147

**Published:** 2014-07-04

**Authors:** Ashutosh Wadhwa, Rachel E Johonson, Keiko Eda, W Ray Waters, Mitchell V Palmer, John P Bannantine, Shigetoshi Eda

**Affiliations:** 1 Center for Wildlife Health, Department of Forestry, Wildlife and Fisheries, University of Tennessee Institute of Agriculture, 274 Ellington Plant Science Building, Knoxville, TN 37996, USA; 2 Infectious Bacterial Diseases of Livestock Research Unit, National Animal Disease Center, USDA Agricultural Research Service, Ames, IA 50010, USA

**Keywords:** Bovine tuberculosis, Cattle, White-tailed deer, ELISA, *Mycobacterium bovis*, EVELISA

## Abstract

**Background:**

The use of serological assays for diagnosis of bovine tuberculosis (TB) has been intensively studied and use of specific antigens have aided in improving the diagnostic accuracy of the assays. In the present study, we report an in-house enzyme linked immunosorbent assay (ELISA), developed by using ethanol extract of *Mycobacterium bovis* (*M. bovis*). The assay, named (ethanol vortex ELISA [EVELISA]), was evaluated for detection of anti- *M. bovis* antibodies in the sera of cattle and white-tailed deer.

**Methods:**

By using the EVELISA, we tested sera obtained from two species of animals; cattle (n = 62 [uninfected, n = 40; naturally infected, n = 22]) and white-tailed deer (n = 41 [uninfected, n = 25; naturally infected, n = 7; experimentally infected, n = 9]). To detect species specific molecules, components in the ethanol extract were analyzed by thin layer chromatography and western blotting.

**Results:**

Among the tested animals, 77.2% of infected cattle and 87.5% of infected deer tested positive for anti- *M. bovis* antibody. There were only minor false positive reactions (7.5% in cattle and 0% in deer) in uninfected animals. *M. bovis* -specific lipids and protein (MPB83) in the ethanol extract were detected by thin layer chromatography and western blotting, respectively.

**Conclusion:**

The results warrant further evaluation and validation of EVELISA for bovine TB diagnosis of traditional and alternative livestock as well as for free-ranging animal species.

## Background

*Mycobacterium bovis* (*M. bovis*), a member of the *Mycobacterium tuberculosis* complex, causes bovine tuberculosis (TB) [1,2], a zoonotic disease of animals including livestock, alternative livestock (e.g., captive cervids), zoo and wildlife. The major wildlife reservoirs of *M. bovis* include Eurasian badger (*Meles meles*), brushtail possums (*Trichosurus vulpecula*), Eurasian wild boar (*Sus sucrofa*) and white-tailed deer (*Odocoileus virginianus*). For many developed nations, difficulties in eradicating bovine TB often arise due to the presence of infection in wildlife reservoirs with spillover to domestic species and movement of infected animals to bovine TB free regions. Within the US, eradication of bovine TB is hampered by importation of TB cattle from Mexico, the presence of a wildlife reservoir (i.e., white-tailed deer in Michigan), and continued detection of small nests of infection within the domestic herd. Particularly problematic for antemortem testing strategies is the detection of bovine TB in a very small number of animals in large dairy herds.

Bovine TB control relies primarily on detection of TB-affected herds by slaughter surveillance and antemortem testing. While current testing strategies are generally reliable at the herd level, the development of new diagnostic strategies for effective control of bovine TB is urgently needed, particularly for movement or border tests to identify individual infected animals [3]. In a recent report from the United States Animal Health Association, a key recommendation to USDA, APHIS, Veterinary Services was to assist biologics companies in the development and evaluation of improved bovine TB diagnostic tests, emphasizing both stakeholder and federal government support for the development of improved bovine TB testing strategies [4].

The diagnostic assays currently available for detection of bovine TB in cattle and wildlife species could be divided mainly into 2 categories; direct assays (i.e. bacterial culture, microscopic demonstration of acid-fast bacilli and polymerase chain reaction [PCR]) and indirect assays (i.e. detection of cellular or humoral immune response) [5,6]. Among the direct assays, bacterial culture and PCR are primarily used as postmortem tests as these tests lack the necessary sensitivity for antemortem detection of TB-infected animals and generally rely on detection of the organism within samples collected at necropsy. Cell mediated immune-based assays primarily include the tuberculin skin test (TST) [7] and interferon gamma (IFN-γ) release assays [8,9], although other strategies are under development [10]. In spite of advantages like simplicity of the procedure and comparatively inexpensive reagents, the TST is very labor intensive; requires two visits to the farm (i.e., injection of tuberculin and evaluation of the response 72 hours later) and practically difficult for wildlife species. IFN-γ release assays are comparatively expensive; require overnight delivery of viable blood samples and a trained technical staff [11]. Antibody-based assays for detection of bovine TB have shown promising results due to their short turnaround time, cost effectiveness and flexibility of use. Previous studies used cross-reactive antigens and suffered from lower diagnostic specificity. The emergence of sensitive antibody-based diagnostic tests for detection of bovine TB supports the concept of development of a reliable and easy to use serologic test [12-16].

We have previously reported an enzyme linked immunosorbant assay (ELISA) using ethanol extract of *Mycobacterium avium* subsp. *paratuberculosis* (MAP) to detect anti-MAP antibodies at early stage of Johne’s disease and named the assay ethanol vortex ELISA (EVELISA) [17-21]. We also reported an EVELISA based assay for detection of specific antibodies in the sera of farmed red deer [22]. The objective of the present study was to determine the potential for application of the EVELISA test to detect anti- *M. bovis* antibodies in the sera of infected cattle and free-ranging white-tailed deer.

## Methods

### Cattle samples

A total of 62 sera samples from cattle were obtained from the TB serum bank at US Department of Agriculture – Animal and Plant Health Inspection Service. The samples were obtained from farms in three states in the U.S.: Georgia (n = 40; dairy), Michigan (n = 21; beef) and California (n = 1; dairy). All the samples from Georgia were from a bovine TB-free herd whereas the California and Michigan samples were from *M. bovis*–infected cattle, as determined by mycobacterial culture, histology and IS*6110* PCR techniques (performed at the National Veterinary Services Laboratories, Ames, IA). The 22 samples from Michigan and California received *M. bovis* PPD for Caudal Fold Test (CFT). All the 62 samples in this group were also tested for quantification of cellular immune response using IFN-γ assay and comparative cervical tuberculin (CCT) test. The CCT test involves injection of both *M. bovis* and *M. avium* PPD at 2 different sites on the neck. Serum samples were obtained before or at the time of injection of PPDs for skin testing. Of the total animals tested, 2 and 1 animals were categorized as suspected with CFT and CCT, respectively.

### Deer samples

A total of 41 serum samples from white-tailed deer were obtained from the USDA/APHIS. Twenty five samples were from uninfected animals, 7 samples were from naturally infected animals from Michigan and 9 samples were from animals which were experimentally infected with *M. bovis* as previously described in Waters *et al.*, 2004 [23].

### Preparation of ethanol extract

A virulent strain of *M. bovis* (HC2005T), which was originally isolated from an *M. bovis* infected dairy cow, was cultured in Middlebrook’s 7H9 medium (Becton Dickinson, Cockeysville, MD) with addition of 0.05% Tween 80 (Fisher Scientific, Fair Lawn, NJ), 10% oleic acid-albumin-dextrose-NaCl (Becton Dickinson, Microbiology Systems, Franklin Lakes, NJ) at 37°C. For antigen preparation, *M. bovis* bacilli was harvested from stationary phase cultures, suspended in 80% ethanol at 80 mg wet weight of bacterial/ml and agitated by vortex at room temperature for 2 min, and centrifuged at 10,621 × *g* for 10 minutes to dislodge surface antigens. Extracted *M. bovis* antigen was diluted (1:80) in the ethanol solution and 50 μL of the solution was immobilized on wells of a 96-well microtiter plate (Costar™, Corning, MA) by evaporation.

### EVELISA

The antigen-coated plate was incubated with 150 μL of buffer B (10 mM phosphate buffered saline, pH 7.0 [PBS], containing 0.05 v/v% Tween 20 [Fisher Scientific, Fair Lawn, NJ] and 10 v/v% SuperBlock [Pierce Biotechnology, Rockford, IL]) at room temperature for 30 min. The plate was then washed 4 times with 200 μL of PBST (10 mM PBS [pH 7.0] containing 0.05% Tween 20). Fifty μL of serum sample (preabsorption of cross-reactive antibodies with heat-killed *Mycobacterium phlei* [0.5 mg/mL] for 30 minutes) was then inoculated and incubated at room temperature for one hour. After washing the wells four times with 200 μL of PBST, 100 μL of horseradish peroxidase (HRP)-conjugated goat anti-bovine IgG heavy and light chains (for cattle samples) or 50 μL of horseradish peroxidase (HRP)-conjugated rabbit anti-deer IgG heavy and light chains (for deer samples) (1:1000 dilution; Kirkegaard & Perry Laboratories, Inc. Gaithersburg, MD; diluted in buffer B) was added to each well and incubated at room temperature for one hour. After washing the wells four times with 200 μL of PBST, 100 μL of tetramethylbenzidine (TMB) solution (as suggested by the manufacturer; Thermo Scientific, Rockford, IL) was used to develop color reaction (10 min) according to manufacturer’s instruction and optical density (OD) of the solution was determined by a microplate reader (Model 680, BioRad, Hercules, CA) at 450 nm after terminating the reaction by adding 100 μL of 2 M sulfuric acid.

### Thin layer chromatography

The ethanol extract was dried by spinning in a speed vacuum concentrator (Thermo Fisher Scientific Inc., Waltham, MA) and washed with a mixture of 1 mL of chloroform/methanol (2:1) and 200 μL of water. The organic layer was transferred to a new tube and dried. The extract was then re-suspended in a chloroform/methanol (2:1) solution at the concentration of 5 mg/mL and separated by thin layer chromatography on an aluminum-backed Silica gel plate (TLC Silica gel 60, Merck KGaA, Darmstadt, Germany) with a development solution (chloroform/methanol/water [90:10:1]). The Silica gel plates were then stained using a polymolybdate solution.

### Western blotting

To determine if *M. bovis* specific immunodominant antigen, MPB83, was present in *M. bovis* ethanol extracts, a Western blot using monoclonal antibody against MPB83 (1 F11, kind gift from Dr. Jim McNair, Agri-Food Biosciences Institute, Northern Ireland) was conducted. The extract (~10 μg) and recombinant protein (7 μg) were mixed 1:1 with 2× SDS-PAGE loading dye and loaded onto a 12% (w/v) SDS-PAGE gel for electrophoretic separation. Electro-transfer of proteins onto pure nitrocellulose was accomplished with the Bio-Rad Trans Blot Cell (Bio-Rad Laboratories) with sodium phosphate buffer (25 mM, pH 7.8) at 0.8 A for 90 min. After transfer, nitrocellulose was blocked with phosphate-buffered saline (PBS; 150 mM NaCl, 10 mM NaPO4, pH 7.4) plus 2% bovine serum albumin (BSA) and 0.1% Tween-20 (block buffer). Monoclonal antibody was diluted 1:500 in block buffer and incubated on the blot at room temperature for 2 h. After three washes in PBS plus 0.1% Tween-20, blots were incubated for 1.5 h in anti-mouse-peroxidase (Thermo Scientific) diluted 1:20,000 in block buffer. The blots were again washed three times as described above and developed using SuperSignal West Pico chemiluminescent substrate (Thermo Scientific).

### Statistical analysis

All experiments were conducted in duplicate or triplicate, and repeated at least twice. The test sensitivity was determined by dividing the number of *M. bovis* culture- test positive animals by the total number of *M. bovis* culture positive animals, with the result expressed as a percentage. The test specificity was determined by dividing the number of bTB free, test-negative animals by the total number of bTB free animals, with the result expressed as a percentage. The cut-off value was determined based on the two-graph receiver operating characteristic (ROC) analysis [24].

### Ethical approval

All animal care and use procedures were reviewed and approved by the NADC Animal Care and Use Committee.

## Results and discussion

**Figure 1:**
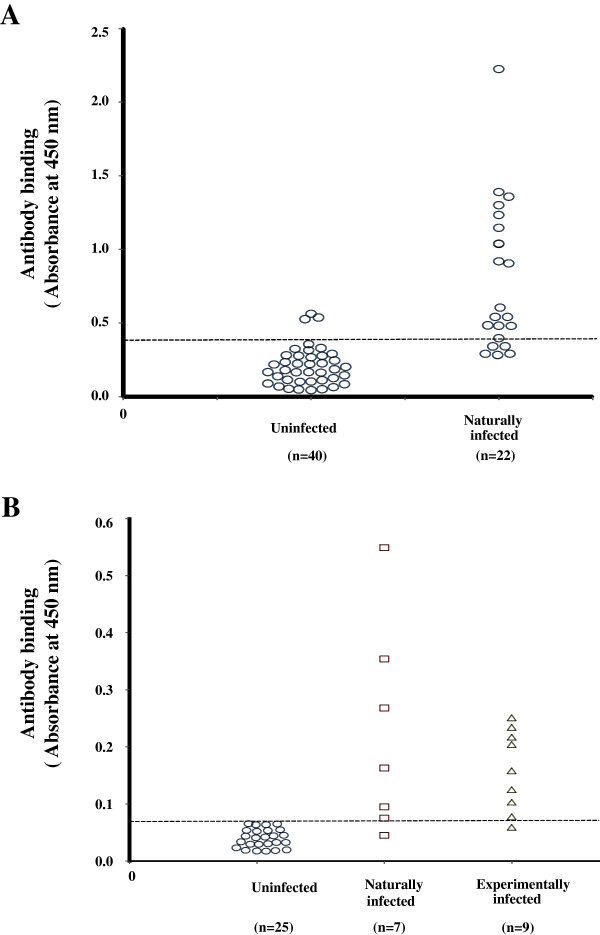
**A - Ethanol-vortex enzyme linked immunosorbent assay (EVELISA).** Antibody binding on serum samples from uninfected (n = 40) and naturally infected (n = 22) cattle were tested by EVELISA test. All the uninfected animals were from a bovine TB free herd and the naturally infected animals were determined as infected by mycobacterial culture, histology and IS*6110* PCR techniques. A cut-off value of 0.4 was used to distinguish *M. bovis* negatives and positive animals. **B** - EVELISA results from sera of deer: Antibody binding in serum of uninfected (n = 25), *M. bovis* -infected – naturally (n = 7) and experimentally (n = 9) deer was determined using the EVELISA test. A cut-off value of 0.068 was used to distinguish between *M. bovis* -negative and positive animals. Both the cut-off values were determined to maximize the sum of sensitivity and specificity values. All experiments were conducted in duplicate or triplicate and repeated at least twice. The statistical difference of antibody binding was evaluated using Mann–Whitney *U* test.

**Figure 2:**
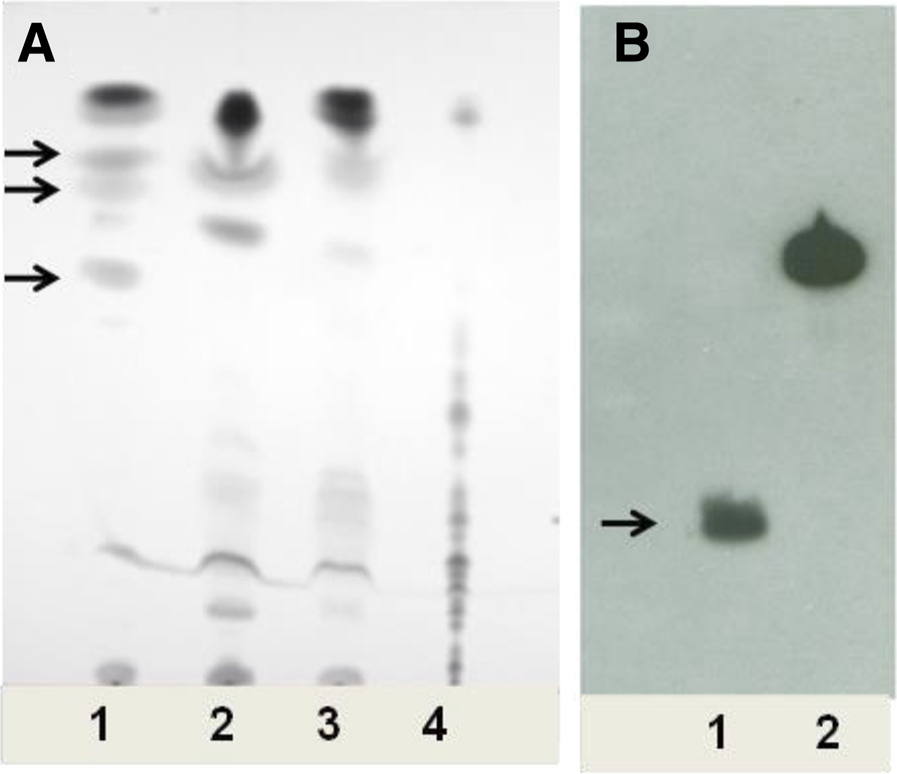
**Thin layer chromatography (TLC) (A) and immunoblot (B) of ethanol extracts of mycobacterial isolates*. *(A)** Dried ethanol extracts of mycobacterial isolates were Folch washed and then developed on a silica-gel TLC plate using a mixture of Chloroform: Methanol: Water (90:10:1) to detect the presence of species specific molecules in the extract of *M. bovis*. The plates were sprayed with polymolybdate solution and the molecules were heated prior to visualization. Ethanol extracts of *M. bovis*, MAP (K10 Strain), MAP (Linda Strain) and *M. avium* subsp. *avium* were used in the lanes 1, 2, 3 and 4, respectively. **(B)** The MPB83 protein is present in the *M. bovis* ethanol extract. Proteins in *M. bovis* ethanol extract were separated by SDS-PAGE and transferred on to a nitrocellulose membrane. An anti-MPB83 monoclonal antibody labeled both the native MPB83 in the ethanol extract (lane 1, arrow) and the recombinant MPB83 fusion protein (lane 2, arrow).

A total of 62 serum samples from uninfected (n = 40) and *M. bovis* infected (n = 22, natural infection) beef - dairy cattle were used to test in the EVELISA format to detect anti- *M. bovis* antibodies (Figure 1A). Thirty-seven (92.5%) of the *M. bovis* uninfected animals which were obtained from TB-free herds showed lower levels of antibody binding than the tentative cut-off value of 0.4. Of the 22 serum samples obtained from bovine TB positive animals, 17 samples (77.3%) showed higher antibody binding than the cut-off. Of the 5 bovine TB positive animals with antibody binding lower than the cut-off, three animals had negative IFN-γ assay results and the other two had CCT “suspect” test results. Forty one serum samples from deer were tested to detect the level of anti- *M. bovis* antibodies in EVELISA format (Figure 1B). All of the samples from uninfected animals (n = 25) showed a lower level of antibody binding than the tentative cut-off value of 0.068. The cut-off value as determined by ROC analysis was much lower than that of cattle, which may be due to lower antibody titer in deer than in cattle or lower affinity of anti-deer IgG secondary antibody than that of anti-bovine IgG antibody. All the samples from the bovine TB positive deer except 1 animal each in the naturally (n = 7) and experimentally (n = 9) infected groups showed higher levels of antibody binding than the cut-off. In this study, *M. phlei* was used to preabsorb cross-reactive antibodies in the serum samples. The preabosorption improved accuracy of EVELISA when compared to that of EVELISA with no-absorption (data not shown). However, the cause of cross-reactive antibodies in EVELISA with no-absorption is not known. The presence of *M. bovis* -specific molecules in the ethanol extract of *M. bovis* was analyzed by thin layer chromatography (TLC) and Western blotting. The TLC lipid analysis suggested the presence of three *M. bovis* -specific lipids in the extract after staining with polymolybdate solution (Figure 2A; Lane 1, Arrows) when compared with lipid profile of ethanol extracts from MAP (K10 Strain), MAP (Linda Strain) and *M. avium subsp. avium* (Figure 2A; Lane 2, 3 and 4). By Western blot analysis, MPB83 was detected in the ethanol extract (Figure 2B). Since MPB83 was shown to be antigenic and react with antibodies in *M. bovis* -infected animals, it is likely that the molecule was contributing to the antibody reaction in the EVELISA test used in this study. On the other hand, the specific molecules detected by TLC may not have contributed to the antibody reaction in EVELISA because antibody reaction was low when chloroform-extractable fraction of the ethanol extract was tested in an ELISA format (data not shown).

Recent advances with use of *M. bovis* specific proteins such as MPB83, MPB70, ESAT-6, and CFP-10 have improved the specificity of serologic-based assays; however, with only minimal improvement of sensitivity [5]. Several studies have used these antigens and evaluated diagnostic sensitivity and specificity. For example, De Anda *et al.*, 1996 reported a sensitivity of 73% using MPB70 in an ELISA format to test 120 *M. bovis* infected and 223 cattle from bovine TB free herd [25]. Waters *et al.*, 2011 tested MPB83 and MPB70 using sera from infected cattle and reported apparent sensitivity and specificity of 63% and 98%, respectively [16]. ESAT-6 and CFP-10 were used in an ELISA format by Whelan *et al.*, 2008 using a total of 1489 bovine TB negative and 522 bovine TB positive cattle sera and stated sensitivity of 40.6% - 86.6% and specificity of 82.6% - 69.7%, based upon use of two different test interpretation criteria [26]. In the present study, we detected anti- *M. bovis* antibodies in 77.2% and 87.5% infected cattle and deer, respectively. However, the number of samples in this study is not enough to compare diagnostic accuracy and there is a need to test more samples to further validate the EVELISA for use in the diagnosis of bovine TB.

## Conclusions

Control activities for bovine TB are being regularly discussed by the regulatory agencies since the organism has been recognized as a threat to the One Health triad (human-livestock-wildlife). Serological assays with their logistics and financial advantages have proved to fit very well in the strategy for controlling bovine TB. Our data indicated that EVELISA test may have a diagnostic sensitivity comparative with ELISA tests reported previously and warrant further studies using a larger number of samples to validate the accuracy of the test. We recently showed that serodiagnosis of Johne’s disease with ethanol extract of MAP can be done in two minutes by a capacitance biosensor which may be a potential format for development of an on-site test for bovine TB using the *M. bovis* extract [27,28].

## Abbreviations

TB: Tuberculosis; *M. bovis*: *Mycobacterium bovis*; MAP: *Mycobacterium avium ssp. paratuberculosis*; ELISA: Enzyme linked immunosorbent assay; EVELISA: Ethanol vortex enzyme linked immunosorbent assay; TST: Tuberculin skin test; CCT test: Comparative cervical tuberculin test; PPD: Purified protein derivatives; PCR: Polymerase chain reaction; IFN-γ: Interferon gamma.

## Competing interests

The authors declare that they have no competing interests.

## Authors’ contribution

SE conceived the study, carried out the statistics and designed the experiments. AW, REJ, KE, JPB conducted the experiments. AW drafted the final manuscript with the help of SE. WRW, MVP and JPB helped in designing the experiments and provided samples – reagents for the study. All authors read and approved the final manuscript.

## Author’s information

AW was a graduate student (when the study was conducted) and SE an Associate Professor in the Center for Wildlife Health, Department of Forestry, Wildlife and Fisheries, University of Tennessee Institute of Agriculture, Knoxville, Tennessee. REJ is a Veterinary Student at the College of Veterinary Medicine, University of Tennessee, Knoxville. KE is a Research Coordinator in the Center for Wildlife Health, Department of Forestry, Wildlife and Fisheries, University of Tennessee Institute of Agriculture, Knoxville, Tennessee. WRW and MVP are Veterinary Medical Officers and JPB is a Research Microbiologist at USDA, Ames, IA.
